# Spin filtering by proximity effects at hybridized interfaces in spin-valves with 2D graphene barriers

**DOI:** 10.1038/s41467-020-19420-6

**Published:** 2020-11-09

**Authors:** Maëlis Piquemal-Banci, Regina Galceran, Simon M.-M. Dubois, Victor Zatko, Marta Galbiati, Florian Godel, Marie-Blandine Martin, Robert S. Weatherup, Frédéric Petroff, Albert Fert, Jean-Christophe Charlier, John Robertson, Stephan Hofmann, Bruno Dlubak, Pierre Seneor

**Affiliations:** 1 Unité Mixte de Physique, CNRS, Thales, Université Paris-Saclay, 91767 Palaiseau, France; 2 Institute of Condensed Matter and Nanosciences (IMCN), Université Catholique de Louvain, B-1348 Louvain-la-Neuve, Belgium; 3 Department of Engineering, University of Cambridge, Cambridge, CB21PZ UK; 4 School of Chemistry, University of Manchester, Oxford Road, Manchester M13 9PL UK; 5 University of Manchester at Harwell, Diamond Light Source, Didcot, Oxfordshire OX11 0DE UK

**Keywords:** Engineering, Materials science, Nanoscience and technology, Physics

## Abstract

We report on spin transport in state-of-the-art epitaxial monolayer graphene based 2D-magnetic tunnel junctions (2D-MTJs). In our measurements, supported by ab-initio calculations, the strength of interaction between ferromagnetic electrodes and graphene monolayers is shown to fundamentally control the resulting spin signal. In particular, by switching the graphene/ferromagnet interaction, spin transport reveals magneto-resistance signal MR > 80% in junctions with low resistance × area products. Descriptions based only on a simple K-point filtering picture (i.e. MR increase with the number of layers) are not sufficient to predict the behavior of our devices. We emphasize that hybridization effects need to be taken into account to fully grasp the spin properties (such as spin dependent density of states) when 2D materials are used as ultimately thin interfaces. While this is only a first demonstration, we thus introduce the fruitful potential of spin manipulation by proximity effect at the hybridized 2D material / ferromagnet interface for 2D-MTJs.

## Abstract (web summary)

2D materials are foreseen as an opportunity to tailor spintronics devices interfaces, a.k.a spinterfaces. Here, using state-of-the-art large-scale integration in spin-valves, authors demonstrate that hybridization of graphene with a metallic spin source results in strong spin filtering effects.

## Introduction

Spintronics has demonstrated its potential with widely distributed applications such as hard disk drives, leading to the big data era. Its flagship application is now magnetic random access memories (MRAMs) based on magnetic tunnel junctions (MTJs) toward faster and greener electronics^1,2^. Introducing novel 2D materials in MTJs is particularly attractive as these materials define sharp interfaces with unique properties, and the performance of spintronics devices is heavily dependent on interfacial properties. However, vertical spin transport through 2D materials has only just started to be studied. The role of many experimental parameters remains yet to be understood. There have been several initial reports on the behavior of spintronic devices incorporating 2D materials^3–7^ (e.g. ultra-small resistance × area products, strong spin-filtering effects, barrier against oxidation/diffusions, etc.), but many experimental parameters are still to be studied in these systems. The expanding 2D materials family is now the focus of several on-going vertical spin transport studies, taking advantage of, for instance, tunneling (h-BN, etc.)^8,9^, semiconducting (MoS_2_^10,11^, WS_2_^12,13^, etc.) and magnetic (CrI_3_^14^, etc.) behaviors. Interestingly, a phenomenon of “bulk” K-point spin filtering (due to a fortunate match between graphene Dirac points and transition metal ferromagnets (FM) minority spin bands) has been early described by Karpan and co-workers^15^. Experimental exploration of this phenomenon has started (see review^3^) with interesting fundamental and technological consequences: for instance ref. ^16^ discusses K-point spin filtering with a weak spin-splitting of the Dirac cone and shift of the Fermi level due to charge transfer. However, a major difficulty concerns the integration of these 2D materials with FM without deteriorating their properties. While many 2D-based heterostructures can be readily fabricated through exfoliation or wet transfer techniques, these ambient processes usually result in degraded interfaces with reduced spin polarizations. Illustratively, the tunneling magnetoresistance (MR) values obtained for FM/monolayer Gr/FM MTJs structures have so far only reached 3.4%, most probably as a result of these integration issues^4,16–19^. A solution has been found with direct chemical vapor deposition (CVD) that has shown to provide clean and well-defined interfaces, but its exploration is still at its infancy as it requires the processes to be carefully adapted to each particular system^20–23^.

**Fig. 1:**
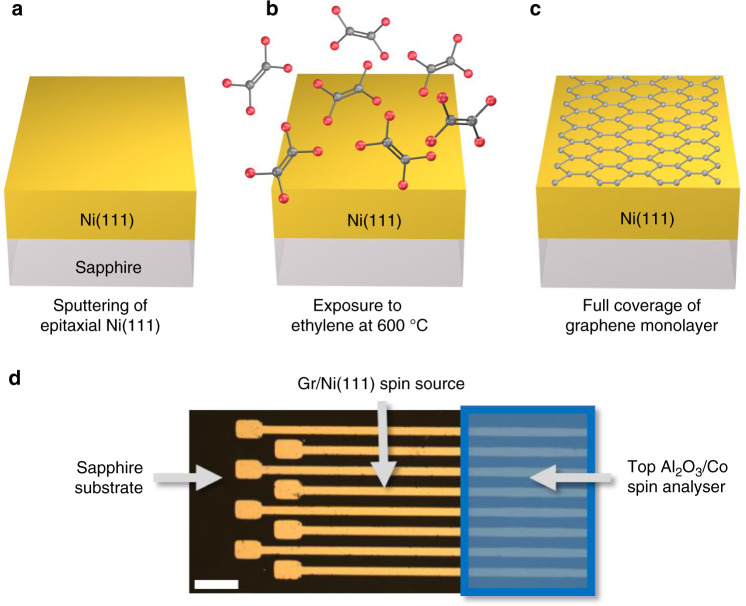
Schematic illustration of the main steps for the Ni(111)/graphene spin source fabrication. **a** Ni(111) is epitaxially grown by a sputtering step at 600 °C on sapphire substrate. **b** The Ni(111) film is then exposed to a low pressure 600 °C CVD step using ethylene as a carbon precursor. **c** This results in the full coverage of the Ni(111) electrode with an epitaxial graphene monolayer. **d** Optical image of the patterned Ni(111)/graphene spin source (scale bar corresponds to 200 µm). A top Al_2_O_3_/Co spin analyzer is further deposited on patterned micro-junctions.

**Fig. 2:**
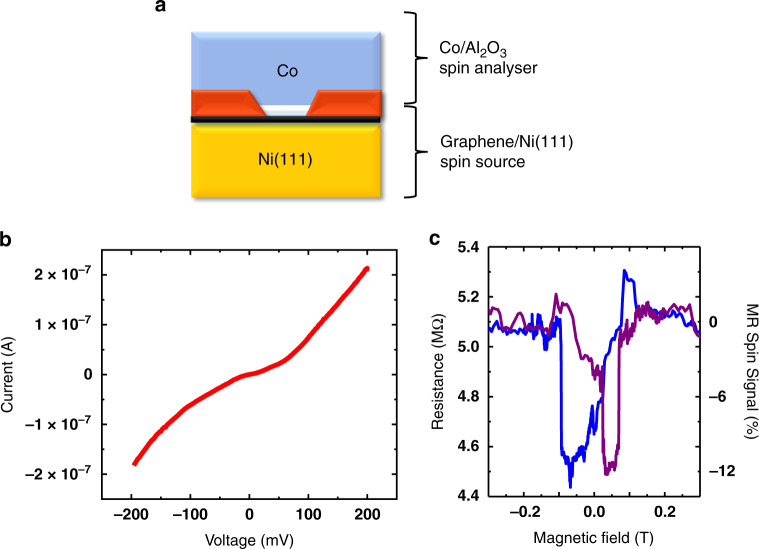
Analysis of the spin properties of the Ni(111)/graphene electrode. **a** Scheme of the full device configuration. After the opening of small micrometric junctions by a lithographic step, top reference Al_2_O_3_/Co spin analyzer is deposited by ALD followed by sputtering. **b** Non-linear dc *I–V* characteristics show the expected tunneling behavior of the Al_2_O_3_/Co spin analyzer and **c** magneto-transport dc measurements reveal a −12% TMR spin signal. The epitaxial Ni(111)/graphene electrode is thus shown to produce a strong spin filtering effect.

**Fig. 3:**
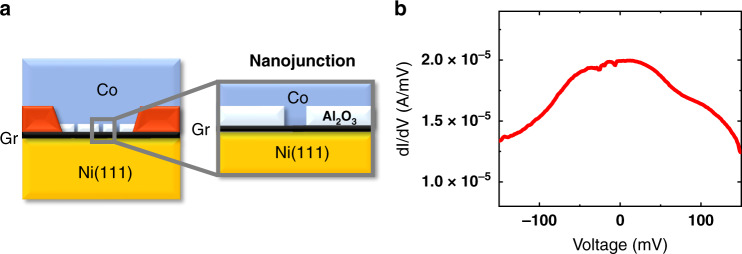
Alternative Al_2_O_3_ growth. **a** Changing ALD growth parameters leads to the reduction of the wetting of the Al_2_O_3_ film on graphene. **b** This translates to a clear signature in transport measurements as shown by the disappearance of tunneling behavior in the d*I*/d*V* (lock-in ac + dc measurements). A direct graphene/Co contact is thus defined over a small effective area, allowing the Ni(111)/graphene/Co spin transport properties to be probed.

**Fig. 4:**
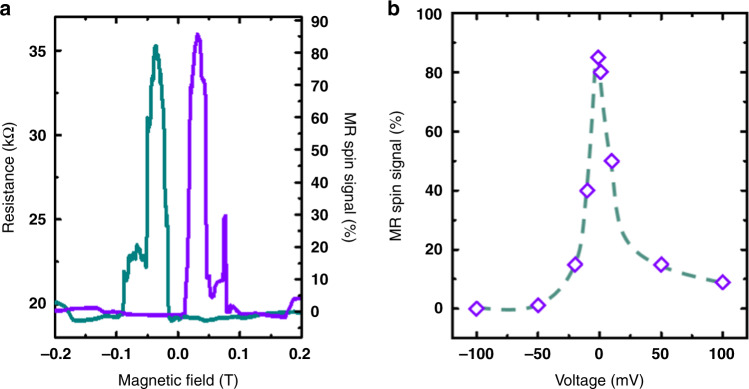
Magneto-transport dc measurements of the Ni(111)/graphene/Co junction. **a** A strong spin signal with MR ~ +80% is measured. **b** Bias dependence of the spin signal is displayed.

**Fig. 5:**
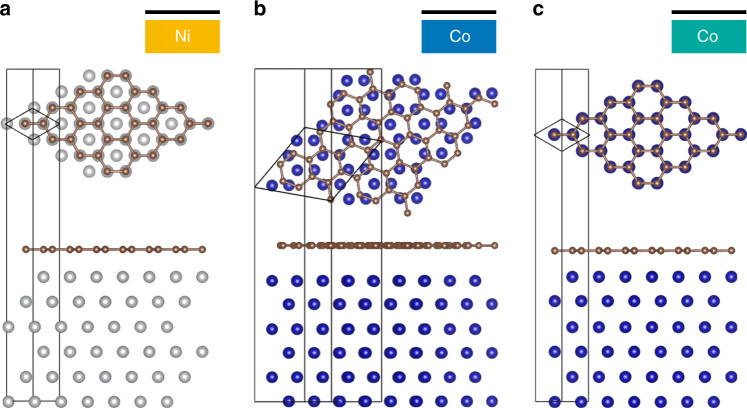
Geometries of the interfaces considered in calculations. **a** Lowest energy epitaxial Ni/Gr interface. For the Co/Gr interface, two cases have been studied. **b** A rotated Co/Gr interface, exhibiting a weak interaction similar to that expected for the low temperature evaporation of a top Co electrode on graphene, and **c** a strong epitaxial interaction. Ab-initio calculated layer separation for the three configurations: **a** Ni–Gr = 2.2 Å, **b** Co–Gr = 3 Å, and **c** Co–Gr = 2.15 Å.

**Fig. 6:**
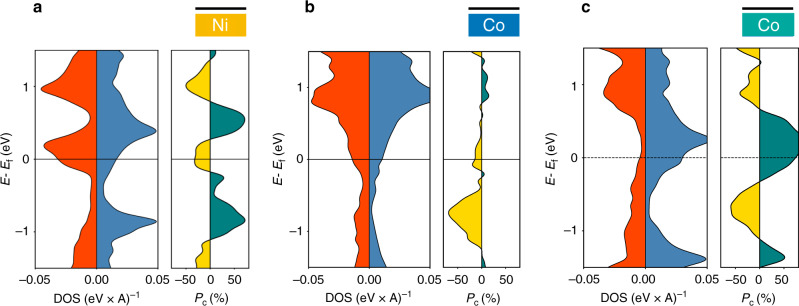
Spin-dependent band calculations for different electrodes taken independently. In all panels, the density of states (DOS) projected on graphene is represented in blue (red) for majority (minority) spins plotted against energy (*E*) relative to Fermi energy (*E*_F_). Positive (negative) spin polarization (*P*_c_) of the hybridized/proximitized graphene layer is depicted in turquoise (gold). First, isolated interfaces alone have been calculated for **a** Ni/Gr, **b** Co/Gr, and **c** Co/Gr with a strong epitaxial interaction. Negative spin polarizations are derived in **a** and **b** cases, as expected from theoretically described spin filtering effects at FM/Gr interfaces. This is in-line with the negative spin polarization measured from Fig. 2 where Ni(111)/graphene is isolated from Co by the Al_2_O_3_ tunnel barrier.

**Fig. 7:**
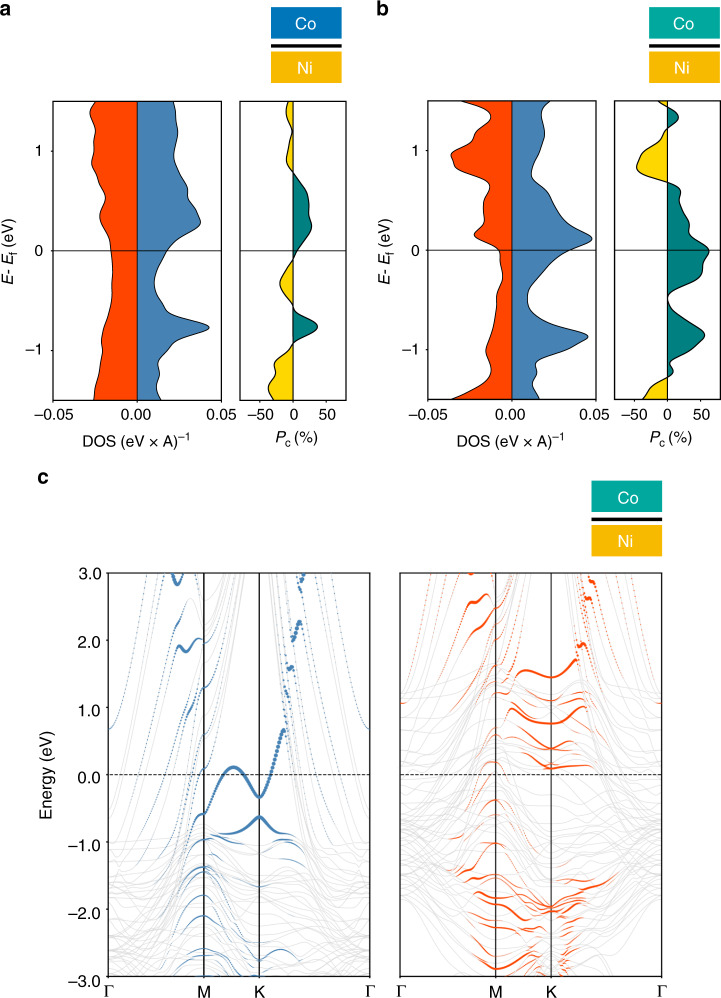
Spin-dependent band calculations for complete spin valve stacks. Strikingly, when **a** both Ni and Co are in contact with graphene, the spin polarization of graphene can be reversed and becomes positive. The amplitude of the spin polarization seems to depend on the extent of FM/Gr hybridization, with a strong increase **b** in the case of strong interaction with Co. This reveals that the spin polarization of graphene in Ni/Gr/Co cannot be simply derived from the spin polarization of graphene in Ni/Gr and Co/Gr. **c** Electronic band structure of Ni/Gr/Co junction for (blue) majority and (red) minority electronic bands are displayed. Blue and red dots correspond to the projection of the majority and minority Hamiltonian eigenstates on carbon atoms. The size of the dot is proportional to the amplitude of the projection, i.e. the “carbon” character of the eigenstate. For majority carriers, the Dirac cone structure is shifted and a gap is opened. In contrast, for minority carriers, the Dirac cone structure has completely vanished due to hybridization. A strong spin asymmetry has been induced by proximity effect.

In this work, we focus on the epitaxial Ni(111)/monolayer graphene system defined by a direct CVD step on a well-crystalized Ni(111) electrode (Fig. 1). We present experiments and supporting first-principles calculations where enabling or disabling the hybridization of graphene with a FM electrode is shown to drastically change the spin polarization and overall leads to large spin signals. Hence an “interfacial” spin-filtering effect is introduced, due to strong hybridization (by proximity effect) of graphene in contact with FM. This effect is concentrated at the interface with a strong impact on the monolayer graphene system, in contrast to previously discussed bulk-related effects^15^. In particular, we fabricate complete MTJs where the potential of graphene as a spin filtering interface is studied by magneto-transport measurements. In between the Co top spin analyzer and the graphene layer, we introduce an interfacial Al_2_O_3_ layer either well-wetted or just below the wetting limit to modulate the Co/graphene interface from a tunnel to a metallic contact. The fabricated Ni(111)/Gr/Al_2_O_3_/Co junctions with a tunnel Al_2_O_3_/Co analyzer reveal a negative MR = −12%, characteristic of Ni/graphene spin filtering (Fig. 2). Conversely, measurements of MR in the metallic Ni(111)/Gr/Co junctions (Fig. 3) show a large MR > 80%, with a positive sign expected from the product of two Gr/FM interfaces with negative spin-polarizations (Fig. 4). Below, we discuss how the amplitude of this spin signal cannot result solely from “bulk” band structure spin filtering as introduced in pioneering ref. ^15^ and that it deviates from a simple Jullière analysis^24^. The modulation of the coupling, achieved here by the tuning of the ultra-thin tunnel barrier in-between the graphene layer and the FM, highlights that simple descriptions based only on K-point filtering (i.e. MR increase with the number of layers^15^) are not sufficient to predict the behavior of our devices. Spin-dependent hybridization, required to understand the strong spin signal measured in our Ni(111)/Gr/Co MTJs, is further discussed in light of first-principles calculations (Figs. 5 and 6). We identify interfacial hybridization as a fundamental process defining the effective spin polarizations. This shows that while “bulk” graphene band structure effects have a profound impact for 2D-based MTJs, they do not catch the whole physics of the interface (Fig. 7). Hybridization interfacial mechanism has consequences that in some cases, as the one shown here, can remarkably outweigh K-point filtering in terms of spin polarization. Our study more generally highlights the potential of 2D materials for spin polarization tailoring in MTJs.

## Results

In Fig. 1, we present the initial fabrication of the epitaxial Ni(111)/monolayer graphene electrode. Ni(111) (80 nm) is epitaxially grown by sputtering on monocrystalline sapphire (0001) at 600 °C. The high crystallinity of the resulting layer is checked by XRD and RHEED measurements^22^. The Ni(111) layer is patterned to define stripes using laser lithography and dry ion beam etching with ionized Ar gas. Monolayer graphene is then epitaxially grown by CVD on top of the Ni(111) electrodes in a low-pressure customized cold-wall reactor using ethylene at 600 °C. The process consists of a pre-annealing step in H_2_ followed by ethylene exposure at 10^−5^ mbar and a cooling step in vacuum. Further details and analysis of the CVD process used for graphene growth on nickel are given in “Methods” section following refs. ^20–22,25,26^. We have made in-depth reports on the quality of graphene in these systems and our study successfully relies on these results. As measured by Raman, and other techniques (in-situ/in-operando and ex-situ XPS, LEED, STM, AFM, Auger, etc.), the graphene in use is of high quality and homogeneity, and epitaxial on the Ni(111) underlying structure. The preservation of the chemical state of the Ni surface against oxidation diffusion has been confirmed by XPS^27^. Junctions are defined on the Ni(111)/Gr stripes by creating small openings (about 10 µm²) in a UVIII resist layer by laser lithography. A thin Al_2_O_3_ layer of 1 nm is deposited using a customized atomic layer deposition (ALD) growth system, directly integrated into a spintronic material deposition cluster. Following refs. ^5,10^ cycles of trimethylaluminum (TMA) and ozone are delivered at 80 °C. We have studied carefully the conditions for the wetting of ultrathin alumina on graphene, see our previous works in particular^5,27,28^ with similar problematics on different 2D materials^29^. For a first set of samples, a well-wetted ALD alumina tunnel barrier is deposited using the previously demonstrated ozone process on graphene (see “Methods” section)^5^. For another set of samples, we decrease the wetting in order to grow a slightly discontinuous Al_2_O_3_ film, without changing other fabrication parameters. This allows a direct contact to the graphene layer over a drastically reduced surface compared to the UVIII window size, aiming at a high enough junction resistance. The Co (15 nm) ferromagnetic top electrode is finally evaporated on the micrometric junctions and capped with Au (80 nm). In summary, we work here with monocrystalline fcc Ni(111) with epitaxially grown graphene and a top contact of amorphous alumina barrier and polycrystalline Co with nm-scale domains. After junction bonding to a measurement chip, we use a low-noise AC + DC transport setup at 4 K to characterize the spin valves, using a Signal Recovery 5210 lock-in amplifier, two Kethley K2182A nanovoltmeters, and a Yokogawa 7651 source connected to the junction.

In Fig. 2, we first focus on the transport measurements of the Ni(111)/graphene/Al_2_O_3_/Co junctions that incorporate a continuous 1 nm Al_2_O_3_ tunnel barrier (Fig. 2a). The *I*(*V*) measurements show the typical tunneling behavior across the Al_2_O_3_ tunnel barrier (Fig. 2b), as observed in previous reports^5,30,31^. This transport behavior, as well as the measured resistance × area values (about 10 MOhms µm^2^), validate the high quality of the ALD-grown tunnel barrier. The top Al_2_O_3_/Co layer can thus be used as a tunnel spin analyzer to probe the spin properties of the Ni(111)/Gr electrode. Magneto-transport measurements through the junction reveal a negative MR = −12% (Fig. 2c). While the negative sign of the MR is expected due to minority spin filtering^3^, its amplitude is doubled compared to previous studies of monolayer graphene junctions with polycrystalline Ni electrodes (−6%) produced using a similar direct CVD process^27^. It also presents a five-fold to ten-fold improvement compared to junctions fabricated with graphene transfer processes^4,17–19^. In previous experimental studies, it has been demonstrated that graphene layers grown on a ferromagnetic polycrystalline Ni electrode by direct CVD are able to induce a spin filtering effect^5,32^, even when only a monolayer graphene is formed^27^. This spin filtering likely results from the predicted band structure matching between Ni minority spins and graphene^15,33,34^, further demonstrated by the experimentally observed increase of spin polarization with the number of graphene layers^3^. This spin filtering effect is however expected to be very sensitive to properties of the ferromagnetic layer in contact^35^. In the framework of analysis introduced by Karpan et al.^15^, this improvement can be related to epitaxial alignment between the graphene and Ni(111), given their closely matched lattice parameters, that results in strong spin filtering. Comparatively, in the polycrystalline case the spin polarization is averaged over many different and less commensurate facets. In this device, with an Al_2_O_3_/Co top tunnel contact, a spin polarization of $P_{{\mathrm{{Ni/Gr}}}}^{{\mathrm{{Tunnel}}}} = - 20\%$ is extracted for the Ni(111)/Gr interface using Jullière’s formula^24,36^ and the previously evaluated $P_{{\mathrm{{Co}}}}^{{\mathrm{{Tunnel}}}} =$ +32% spin polarization of the top Al_2_O_3_/Co tunnel analyzer interface^30,31^.

In Figs. 3 and 4, we present transport measurements in a Ni(111)/Gr/Co junction, where the graphene layer is in direct contact with both FM without tunneling through the alumina. Indeed, in this case as described above, the ALD process to grow the alumina layer is modified to reach the wetting limit. The resulting alumina film is thus slightly discontinuous allowing direct Ni/Gr/Co nanocontacts to be defined (Fig. 3a), thereby adapting to the ultra-low resistance × area product of the Gr interface. The sample fabrication steps remain otherwise identical. The measured d*I*/d*V* response shows a metallic behavior as expected for a direct Ni/Gr/Co contact (Fig. 3b). Strikingly, the magneto-transport measurements reveal a large MR value reaching +82% (Fig. 4a, b). We note that we have observed similar spin signals reproducibly. As a first approximation, we can follow Jullière’s formula to extract an average magnitude of spin polarization at Gr/FM interfaces of |*P*| = 54% in absolute value.

Strikingly, this average spin polarization is well above that of $P_{{\mathrm{{Ni/Gr}}}}^{{\mathrm{{Tunnel}}}} = - 20\%$ extracted by the analysis of the Ni(111)/Gr interface probed with an Al_2_O_3_/Co tunnel spin analyzer. This cannot be explained by the top Gr/Co interface, since the spin polarization of Gr/Co has also been found to be $|P_{{\mathrm{{Gr/Co}}}}^{{\mathrm{{Tunnel}}}}| \, < \, 50\%$ in Co/Al_2_O_3_/Gr/Co structures where Co is on top of graphene (not shown). Thus, for the Ni(111)/Gr/Co device with direct metallic contact, it is reasonable to deduce that the spin polarization of the Ni(111)/Gr interface $|P_{{\mathrm{{Ni/Gr}}}}^{{\mathrm{{Direct}}}}| \, > \, 50\% \gg |P_{{\mathrm{{Ni/Gr}}}}^{{\mathrm{{Tunnel}}}}|$ discussed above as being of about 20% (Fig. 2). This is surprising as the spin polarization of the Ni/Gr interface would be expected to be independent of the nature of the top spin analyzer. This highlights that the MR of this junction is well beyond what can be easily deduced by separate measurements of the spin polarizations of each interface (simple Jullière analysis).

Pioneering theoretical studies have highlighted the strong potential of graphene-based spin valves^15,33,34^. MR up to 33% was expected in the optimistic case for Ni/Gr/Ni monolayer graphene in ref. ^15^. Large MR values were later predicted for the monolayer by ref. ^34^. Interestingly, the >80% MR value falls in the range of values reported by Yazyev and Pasquarello^34^ for fcc Co/Gr/Co (60%), fcc Ni/Gr/Ni (17%), and hcp Co/Gr/Co (86%) junctions based upon quantum transport (QT) calculations. Still, the results of MR for Ni-based junctions are consistently lower: at 17% and 33% versus 60% and 86% for Co-based junctions. Hence one could be surprised that we achieve 82% (which is almost the maximum predicted for Co) while working with a combination of a Ni and Co electrode. But more strikingly, the MR was predicted to drastically increase with the number of layers with up to 10^12^% MR for thick graphene multilayers^15,33^. This pointed to a strong band structure spin filtering effect of graphene—often referred to as a K-point filtering—that shifted the interest towards multilayers. Extensions to this simple K-point spin filtering picture used to interpret the results of the quantum transport calculations of refs. ^15,33^ have also been discussed (see for instance ref. ^37^). Here we focus on the proximity effect related to the monolayer case, where the band structure filtering effect is expected to be minimal. Additional mechanisms must be expected at the interface emanating from the proximity of a ferromagnet with graphene layers. Indeed, proximity effects are expected to play a major role in spin-dependent graphene band structure modifications. It is now well accepted that such hybridization can lead to strong modifications of the interfacial spin properties, as initially proposed more generally for organic materials by Barraud et al.^35^.

The instrumental role of graphene hybridization and its chemical interactions with Ni/Co is confirmed here by first-principles calculations. The spin-dependent properties of the proximitized graphene interlayer are expected to be strongly modulated by the Gr–Ferromagnet layer separation/orientation, but we do not have experimental access to this information. Pioneering theoretical investigations have treated the epitaxial stacking of graphene on Co and Ni FM cases, corresponding to the ground-state and conveniently allowing for small computational cells. However, depending on the fabrication conditions, real devices are likely to present more complex interfaces corresponding to local energy minima. To achieve the closest estimate, we rely on ab-initio calculations of relaxed structures (see Fig. 5) highlighting how different layer separations can be related to different stacking configurations and growth conditions. This demonstrates the necessity of linking experimental conditions and ab-initio calculations in order to better address our system. We believe that the fine tuning of these heterostructures toward larger spin signals will be further guided by fundamental studies in the spirit of pioneering works by Karpan et al.^15,33^ and Yazyev and Pasquarello^34^. The geometries considered in our calculations are illustrated in Fig. 5. Two local energy minima have been considered for the Co(0001)/Gr interface in order to highlight the modulation of the spin-dependent properties of the junction by the strength of interaction with Co. The ground-state configuration is depicted in Fig. 5c. Another locally stable configuration corresponding to a rotated graphene plane is represented in Fig. 5b. We assume the Ni(111)/Gr interface to be in its lowest energy lattice-matched configuration (see Fig. 5a). Slabs of six atomic layers of Co and Ni are used to describe the Ni fcc 〈111〉 and Co hcp 〈0001〉 surfaces. The geometries have been obtained by a full relaxation of the atomic degrees of freedom, the only constraint being that away from the interface the FM match the bulk Ni lattice parameters. Hence the optimized interfaces are locally stable and the computed Ni/graphene and Co/graphene distances correspond to local equilibrium between long-range and short-range interactions. The final atomic structures result from the interplay between long-range van der Waals (vdW) interactions and short-range chemical bonding as accounted for by the vdW-DF functional of Dion et al.^38^ with the exchange modified by J. Klimes et al.^39^ implemented within the SIESTA code^40^. Basis sets of numerical atomic orbitals (double-$\zeta$ + polarization) have been used to expand the wave-functions in all calculations. Integrations in reciprocal space have been performed by means of regular *k*-point grids characterized by a 13.6 Å effective cutoff. The Ni/Gr interface has already been studied in the literature (see for instance refs. ^41–43^) and is presented here as a reference to establish the impact of proximity with the top Co electrode in the full spin valve structure.

The computed spin-resolved projected density of states (PDOS) on carbon atoms for the Ni/Gr and Co/Gr interfaces (Fig. 6) illustrate the destruction of the Dirac cones of the isolated graphene sheet due to its interaction with the FM contact. From the PDOS, we estimate the spin-polarization of the interface as $P_{\rm{{{Gr}}}} = \frac{{\rho _{\rm{{{Gr}}}}^ \uparrow - \rho _{\rm{{{Gr}}}}^ \downarrow }}{{\rho _{\rm{{{Gr}}}}^ \uparrow + \rho _{\rm{{{Gr}}}}^ \downarrow }}$, where $\rho _{\rm{{{Gr}}}}^{ \uparrow \downarrow }$ is the spin resolved DOS projected on graphene. The Ni/Gr epitaxial interface is characterized by a negative spin-polarization around the Fermi level, in agreement with experiments. The spin-polarization of the Co/Gr interface depends on stacking configuration illustrating how different Co/Gr hybridizations give rise to different density of states on the graphene layer. When combined into a full Ni/Gr/Co junction, the computed polarization of the sandwiched graphene plane is found to be positive for both Co/Gr configurations while its magnitude is modulated by the type of hybridization (see Fig. 7a, b). This is a clear manifestation of spin-dependent band structure modifications of the graphene layer due to the proximity (a.k.a. hybridization) of the ferromagnetic electrode, well beyond a simple band structure shift^16^. The proximity effect of the FM on graphene’s electronic structure is further illustrated in Fig. 7c. The projected band structure of the ground-state Ni/Gr/Co junctions highlights the drastic effect of the hybridization which appears strongly spin dependent. The computed projections of the Hamiltonian eigenstates on carbon atoms illustrate the complex spin-dependent hybridization of the graphene sheet with the FM. On the one hand, eigenstates reminiscent of the Dirac cones of pristine graphene, are reported around the K point for low-energy majority carriers. The interaction with the FM is responsible for the opening of an energy gap and an overall shift in energy. On the other hand, no such feature is reported for minority carriers. Low-energy minority carriers in graphene are rather strongly hybridized by proximity effect with Ni/Co eigenstates forming dispersion-less bands around the K point. This proximity effect is governed by the extent of hybridization between graphene and the FM. To emphasize the potential for inducing and modulating graphene’s spin-dependent properties using this proximity effect, we show in Fig. 7b the computed electronic structure of a second Ni/Gr/Co junction characterized by the same Ni/Gr interface but with a different relative orientation of the hcp 〈0001〉 Co surface. The modification of the Co/Gr interface significantly impacts the spin-dependent band structure of the junction and results in a much higher computed spin polarization. Intuitively it is expected that a weaker interaction between the metallic electrodes and the graphene sheet should lead to smaller perturbations of the graphene band structure and hence to a preserved band structure spin filtering allowing larger MR. In contrast, we show here that these band structure modifications can be in some case dramatically beneficial for the selection of a spin direction and their exploitation lead to measured strong spin signal. We believe that further more systematic quantum transport theoretical studies, predictions, and understanding (as previously done in Karpan et al.^15,33^ and more specifically for monolayer in Yazyev and Pasquarello^34^) will enable further clarification and exploitation of this phenomenon.

Alongside our experimental observations, this reveals that spin-dependent band structure modifications of the graphene layer due to the proximity of ferromagnetic electrodes should not be neglected in these ultrathin barrier MTJs. Clearly, the graphene interlayer presents different properties when the interaction with Co is weakened due to the presence of a tunnel barrier, compared to when there is a hybridization resulting from intimate contact with Co. This situation is reminiscent of the case of the 2D insulator h-BN, whose properties have been shown to drastically change, becoming metallic when it hybridizes with a ferromagnetic electrode^9,44,45^. The large MR > 80% measured here in the case of a monolayer graphene-based MTJ illustrates the strong impact of hybridization with ferromagnetic electrodes on the extracted spin polarization. For comparison, previous reports of 2D-MTJs with monolayer graphene showed at best MR = 3.4%^4,17–19^, indicating a very large increase of spin signal output with our direct CVD approach to form epitaxial Gr/FM interfaces. While in our experiments the Co is polycrystalline and the spin polarization is averaged over many facets, we expect even higher TMR spin signals to be obtained if ideal situations like the ones depicted in theoretical studies such as ref. ^34^ can be reproduced. Graphene potential as a spin-filtering interface thus appears very promising, and well beyond initial predictions^15^. Interestingly, beyond our results, we note that many parameters remain uncharted territory in these 2D-MTJs systems. In particular we believe that increased performances and novel functionalities should arise from the study of spin-polarized transport dependence with some of the system parameters, such as the underlying FM crystallography and the related epitaxy/rotation of the graphene layer, the graphene layers stacking sequence and their integration in heterostructures with other 2D materials, etc. This offers a large playground, unseen with conventional oxides, towards more control to tailor spin transport with 2D layers.

## Discussion

Our study exposes the significant potential of 2D hybridization for spin filtering in MTJs. The use of a tunnel analyzer confirms the negative polarization induced by graphene spin filtering at the Ni/Gr interface, with a strong enhancement shown here in the case of an epitaxial interface. Furthermore, the very large MR of >80% measured in a low resistance × area Ni(111)/Gr/Co magnetic tunnel junction highlights that much more can be exploited from monolayer graphene than the already impressive band-structure spin-filtering expected for thicker layers^3,15^. Surprisingly, our values are on par with best figures of works of Karpan et al.^15^ and Yazyev and Pasquarello^34^ for monolayers of Co/Gr/Co and Ni/Gr/Ni and exceed naïve expectations that could be derived for Co/Gr/Ni. It evades simple analysis using the Jullière model^24^. We show here that monolayer graphene spin transport properties are strongly dependent on its hybridization with FM metals which are in close proximity (here top Co). This opens further potential for 2D-MTJs, as such proximity effects on 2D materials provide the opportunity to modify and tune spin properties (in a “spinterface” way^46^) beyond their intrinsic expectations. This additional degree of freedom, illustrated by our present results, offers a rich assortment of 2D material-based MTJ configurations for future theoretical and experimental studies.

## Methods

### Ni(111) electrode definition

A 80 nm Ni(111) layer is epitaxially grown by sputtering on monocrystalline sapphire (0001) in a customized PLASSYS MP-900S system. The growth is carried at 600 °C in a 80 W Ar plasma and at a rate of 1.89 A/s. The resulting film is patterned into stripes using laser lithography with SPR700 resist and dry Ar ion beam etching.

### CVD graphene growth

Monolayer graphene is epitaxially grown by CVD on top of the Ni(111) electrodes in a customized cold-wall reactor whose base pressure is 5 × 10^−7^ mbar. Samples are heated to 600 °C at 300 °C/min in 1 mbar of H_2_ and annealed for 15 min. The H_2_ is then removed and the samples are exposed to a 10^−5^ mbar of C_2_H_2_ at 600 °C. Finally, the samples are left cooling in vacuum at ∼100 °C/min.

### MTJ fabrication

Samples are covered with UVIII resist and small windows of about 10 µm² are opened above the Ni(111)/Gr stripes by laser lithography. A customized BENEQ atomic layer deposition (ALD) growth system, directly integrated into the spintronic material deposition cluster, is used to grow Al_2_O_3_ layers of 1 nm. Depending on the targeted junction (tunnel Al_2_O_3_ layer or direct Co contact), a wetting step is initially carried using a 60 s ozone atmosphere. The Al_2_O_3_ growth is achieved by 10 cycles of TMA and ozone delivered at 80 °C sequentially, with a growth rate of 1 A per cycle. The sample is then partly protected by a shadow mask to preserve bonding pads. Using an e-beam evaporator, a gold capped 15 nm Co ferromagnetic top electrode is deposited on the samples. The devices are then bonded in a ceramic chip for measurements.

## Data Availability

The datasets generated during and/or analyzed during the current study are available from the corresponding author on reasonable request.
